# GraphVelo allows inference of multi-modal single cell velocities and molecular mechanisms

**DOI:** 10.1101/2024.12.03.626638

**Published:** 2024-12-07

**Authors:** Yuhao Chen, Yan Zhang, Jiaqi Gan, Ke Ni, Ming Chen, Ivet Bahar, Jianhua Xing

**Affiliations:** 1. Department of Bioinformatics, College of Life Sciences, Zhejiang University, Hangzhou, 310058, China; 2. Department of Computational and Systems Biology, University of Pittsburgh, Pittsburgh, PA, USA; 3. Laufer Center for Physical and Quantitative Biology, Stony Brook University, Stony Brook, NY, USA; 4. UPCI; 5. Department of Physics and Astronomy, University of Pittsburgh, Pittsburgh, PA, USA

## Abstract

RNA velocities and generalizations emerge as powerful approaches for exacting dynamical information from high-throughput snapshot single-cell data. Several inherent limitations restrict applying the approaches to genes not suitable for RNA velocity inference due to complex transcriptional dynamics, low expression, or lacking splicing dynamics, and data of non-transcriptomic modality. Here, we present GraphVelo, a graph-based machine learning procedure that uses RNA velocities inferred from existing methods as input and infer velocity vectors lie in the tangent space of the low-dimensional manifold formed by the single cell data. GraphVelo preserves vector magnitude and direction information during transformations across different data representations. Tests on multiple synthetic and experimental scRNA-seq data, as well as multi-omics datasets demonstrate that GraphVelo, together with downstream Dynamo analyses, extends RNA velocities to multi-modal data and reveals quantitative nonlinear regulation relations between genes, different layers of gene regulation, and between virus and host cells.

## Introduction

Cells need to constantly detect and respond to changes of extracellular and intracellular environment, and one mechanism of the response is through regulating gene transcription. Multiple factors affect the transcriptional activity of a eukaryotic gene such as cis and trans regulatory elements and chromatin structures. High throughput single cell genomics data provide the landscape of cell phenotypes. These sequencing data, however, miss information on how the genome states change over time due to the destructive nature of the experimental procedures. Continuous efforts have been made to extract information about gene regulation and connecting the cell states to generate temporal sequences of events from the snapshot data. One group of methods for learning dynamics that has received extensive attention is RNA velocity^1^ and related methods. The original RNA velocity method leverages the ratio between nascent and mature transcripts to estimate a vector describing the rate of change in gene expression. This seminal study has inspired numerous methods for improved RNA velocity estimation based on information from splicing^2–6^, metabolic labeling^7,8^, lineage tracing^9^, transcriptional factor binding^10^, etc.

However, the RNA velocity framework has its own inherent limitations. First, none of the RNA velocity estimation methods can be applied to any single cell transcriptomic data without restriction. For example, the splicing-based method does not apply to genes without introns in organisms like prokaryotes or viruses. Erroneous inference of RNA velocities has also been noticed for genes having complex splicing dynamics^11^. Furthermore, it is difficult to estimate RNA velocities of genes with low expression, which excludes most transcription factors. Second, multi-omics sequencing technologies provide multifaceted information on cellular states complementing to the transcriptome modality, but currently there is limited systematic method to extend velocity estimation to other modalities^12,13^.

With the inferred single cell velocities, in practice one often needs to transform single cell data representation, e.g., from a principal component (PC) space to a UMAP representation. Notice that a cell state is represented as a point in a cell state space, and numerous dimension reduction and manifold learning algorithms have been developed for such representation transformation. In comparison, transforming a velocity vector between representations is a nontrivial and new task not explicitly addressed in the single cell field. Even worse, a visually correct vector field does not necessarily imply accurate high-dimensional velocity estimation^14^. La Manno et al. proposed a cosine kernel method that has been adopted since then in most subsequent studies^1^. Li et al. mathematically proved that the cosine kernel asymptotically gives the correct direction of a velocity vector in the large sampling limit, but the magnitude information is completely lost due to a normalization procedure^15^. This loss of information casts concerns when such quantitative information is needed.

In this study, we tackle the above challenges through a graph-theoretical representation of RNA velocities with dynamical systems underpinnings. GraphVelo takes an ansatz that the measured single cell expression profiles and inferred RNA velocities collectively reflect a dynamical process and are connected through a set of dynamical equations. It exploits such additional constraint that couples high dimensional velocity field and single cell state manifold and generalizes to the context of multi-modal single cell data. While the combined expression and velocity information has been widely used to infer cell state transition trajectories, GraphVelo velocities allow downstream analyses such as Dynamo^7^ to extract quantitative information on the causal relations among genes that dictate the cell state transitions. We benchmark the proposed graph framework with simulated and experimental single cell data for a broad scope of downstream analyses.

## Results

### GraphVelo infers manifold-consistent single cell velocity vectors through tangent space projection and transforms between representations through local linear embedding

Consider that the internal state of a cell can be specified by a N-dimensional state vector x, with N≫1 generally. Assume that the temporal evolution of the cell state follows a continuous and smooth curve $x\left(t\right)$ (See supplemental text for further discussions). The instant velocity vector $v\left(x,t\right)=\;\text{d}x\left(t\right)/\text{d}t$ is always tangent to the curve of $x\left(t\right)$ at x. One can generalize to the situation that the trajectories of a swarm of cells form a M-dimensional manifold $𝓜\left(x\right)$ embedded in the N-dimensional state space with M≪N typically, as revealed by high throughput single cell omics data. Then under the ansatz that a velocity vector $v\left(x,t\right)$ dictates the evolution of a state vector $x\left(t\right)$, v must lie in the tangent space of $𝓜\left(x\right)$, denoted as $T_{p}𝓜$ (see more theoretical discussions in Supplemental Text). In general, the RNA velocity vectors inferred from any existing method do not automatically satisfy this tangent space requirement.

**Figure 1.**
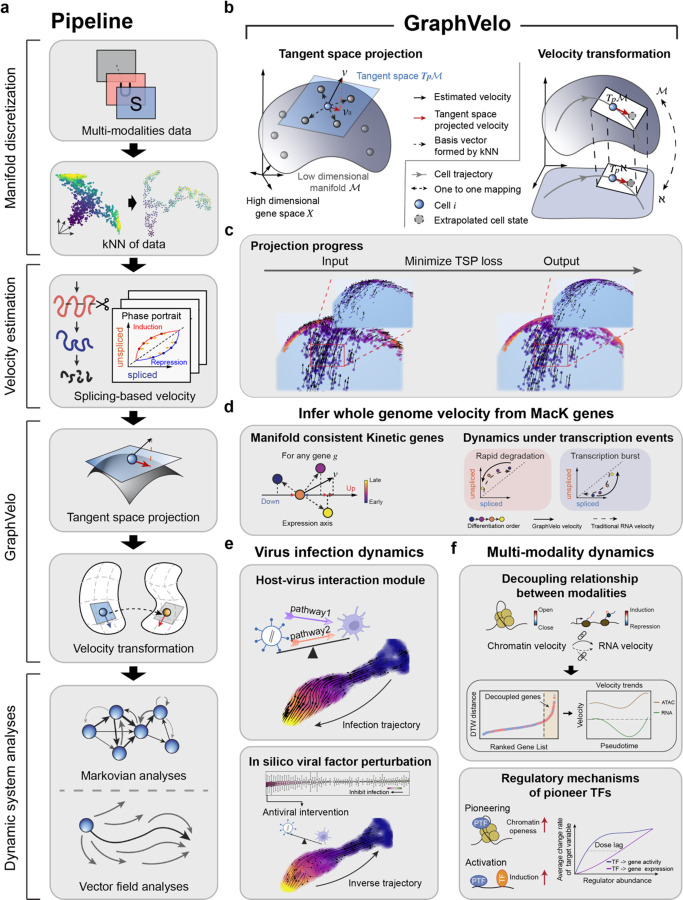
Refining RNA velocity by tangent space projection and transforming between representations using GraphVelo **(a)** Workflow of RNA velocity-based analyses incoporating GraphVelo. **(b)** Schematic of tangent space projection and velocity transformation between homeomophic manifolds. RNA velocity vectors are projected onto the tangent space defined by the discretized local manifold of neighborhood cell samples. **(c)** The progress of minimizing the loss function of tangent space projection. Noisy RNA velocity in an analytical 3d manifold are projected onto the data manifold during optimization. **(d)** GraphVelo allows whole genome velocity inference based on the robustly estimated MacK genes (see also Figure 3). **(e)** Virus infection dynamics and underlying host-virus interaction mechanisms uncovered by GraphVelo (see also Figure 4). **(f)** GraphVelo provides a consistent view of epigenetic and genetic dynamics (see also Figure 5).

Taking various inferred single cell RNA velocity, e.g. splicing-based, metabolic labeling-based, lineage tracing-based, etc., as input, GraphVelo exploits the nature of the low-dimensional cell state manifold to: 1) refine the estimated RNA velocity to satisfy the tangent space constraint on the velocity vectors; 2) infer the velocities of non-transcriptomic modalities using RNA velocities. GraphVelo serves as a plugin designed to seamlessly integrate into existing RNA velocity analysis pipelines, and process single cell data for downstream cellular dynamics analyses using methods such as dynamo (Fig. 1a).

Algorithmically, GraphVelo approximates the tangent space at a cell state x by a k-nearest neighbor (kNN) graph following the local linear embedding algorithm^16^, and uses the more reliable data manifold 𝓜 to refine the velocity vectors by imposing the constraint that the velocity vector v should lie in the tangent space (Fig. 1b). Consider a given point $x_{i}$ on a manifold corresponding to the expression state of sampled cell i. Its infinitesimal neighborhood forms a Euclid space that approximates the tangent space $T_{p}𝓜$. With sufficient sampling of the cells j neighboring to itself in the state space, the displacement vectors between cell i and its neighboring cells, $\delta_{ij}=x_{j}-x_{i}$, form a set of complete albeit possibly redundant and nonorthogonal/non-normalized basis vectors of the Euclidean space in the local region. Then the projection of the measured velocity vector of cell $T_{p}𝓜$ can be expressed as a linear combination,

(1) $$v_{∥}\left(x_{i}\right)=\sum_{j\in {𝓝}_{i}}\phi_{ij}\delta_{ij},$$

where ${𝓝}_{i}$ is the neighborhood of cell i, defined by its k nearest neighbors in the feature space determined by sequencing profiles. Direct application of Eqn. 1 to determine the coefficients $\phi_{\mathrm{ij}}$ is numerically unstable in real data (see Methods for detailed discussion). Instead, we perform the projection by optimizing the tangent space projection (TSP) loss function,

(2) $$𝓛\left(\phi_{i}\right)={\left\|v_{i}-v_{∥i}\right\|}^{2}-b\cdot \cos\left(\phi_{i},\phi_{i}^{corr}\right)+\lambda {\left\|\phi_{i}\right\|}^{2},$$

where $\left\|\cdot \right\|$ refers to vector modulus. $\phi_{i}^{corr}$ is a heuristic “cosine kernel” widely used in the RNA velocity analyses for projecting velocity vectors to a reduce space, with the second term cos(⋅,⋅) denoting the cosine similarity and k the number of neighbors for each cell. The first term in the loss function learns the correctly-scaled velocity magnitudes, and the second term retains the reliable direction information based on previous mathematical analyses showing that $\phi_{i}^{corr}$ asymptotically gives correct direction of the velocity vector^15^. The L-2 regularization is used to bound parameters $\phi_{i}$. b and λ are two hyperparameters determining the emphases on retaining the direction ^*i*^and the regularization, respectively.

With local linear embedding it is straightforward to transform velocity between different representations. Assuming there exists a mapping function f connecting manifold 𝓜 and ℵ such that for cell i with state vector $x_{i}$ in 𝓜, the coordinate of the same cell in ℵ is given as $y_{i}=f\left(x_{i}\right)$. Since a given local patch of continuous manifold is approximated by a Euclidean space, a locally linear transformation connects the patch in the two representations. Consequently, for a vector described by Eqn. 1 in 𝓜, the velocity vector in ℵ is,

(3) $$v_{∥}\left(y_{i}\right)=\sum_{j\in N_{i}}\phi_{ij}{\delta^{\prime }}_{ij},$$

where ${\delta^{\prime }}_{ij}=y_{j}-y_{i}$.

The above equation also applies to transformation between a manifold embedded in a state space and in a subspace. The celebrated Whitney Embedding theorem states that any smooth real M-dimensional manifold can be embedded in a 2M-dimensional real space provided M>0. Consider a full set of genes versus a subset in a scRNAseq dataset, or a combined scRNAseq/scATACseq multiomics dataset versus the scRNAseq subset. Assume that the full cell state space has a dimensionality N, while a single cell data manifold is typically low-dimensional with M≪N. Then the Whitney Embedding theorem^17^ suggests that with proper choice of the subset the manifolds in the full and subspace are homeomorphic or at least piece-wise homeomorphic (Fig. 1b), i.e., a one-to-one mapping exists between the two. Then applying Eqn. 3 allows one to infer the velocity vectors for the full-space representation from those of the subspace.

Therefore, Eqn. 1–3 form the mathematical and computational foundation of GraphVelo. With velocity vectors refined with GraphVelo, one can further perform downstream analyses, as cases exemplified in Fig.1d–f.

### Benchmark studies demonstrate effectiveness of GraphVelo across simulation datasets with diverse topology

**Figure 2.**
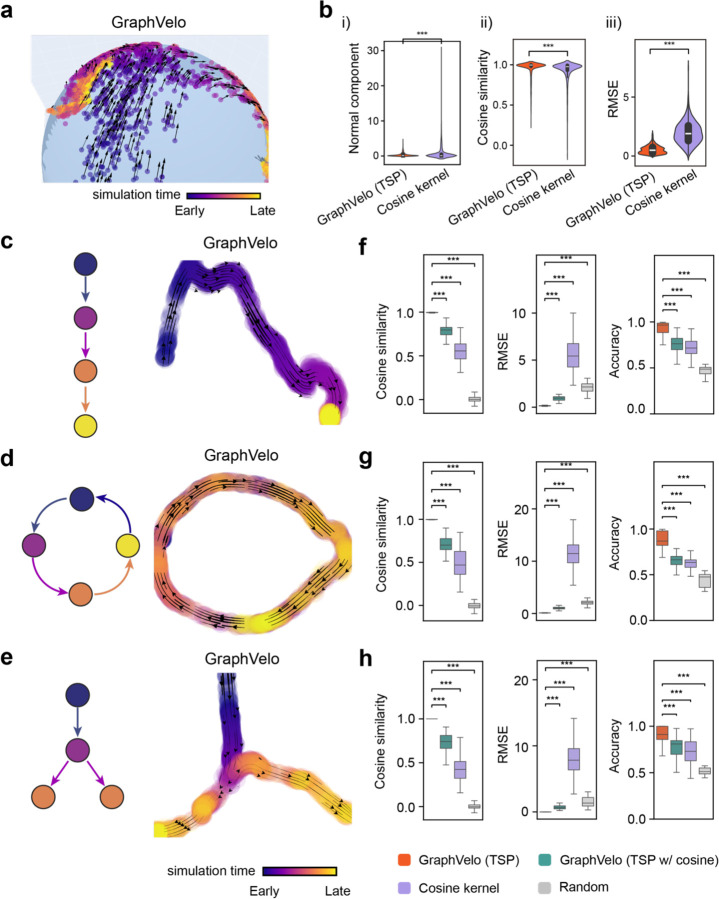
Testing graphVelo on simulated datasets. **(a)** Streamlines of an analytical three variables bifurcating vector field. The cells are colored by simulation time. **(b)** Violinplots of: i) normal component, ii) cosine similarity and iii) root mean square deviation (RMSE) between ground truth and velocity projected by GraphVelo and cosine kernel. **(c-e)** Simulation of scRNA-seq data using dyngen and velocity fields reconstructed using GraphVelo velocities under linear, cycling, and bifurcating differentiation models, respectively. Each simulation consists of 1,000 cells and 100 genes. Cells are colored by their simulation time along trajectory. **(f-h)** Boxplots of cosine similarity, RMSE and accuracy between ground truth and velocity projected by GraphVelo TSP loss without cosine regularization, GraphVelo TSP loss with cosine regularization, cosine kernel and random predictor.

To demonstrate the effectiveness of the geometry constrained projection, we first benchmarked our method on a 3D bifurcation system embedded in a 2D manifold (Methods). We added a random component vertical to the tangent plane to mimic the noise. The resulting velocity vectors inferred by GraphVelo through minimizing the TSP loss were consistent with the ground truth vectors (Fig. 2a). Both GraphVelo and cosine kernel successfully removed the normal components (Fig. 2b i) and maintained the directional information (Fig. 2b ii), but only GraphVelo kept the velocity magnitude information (Fig. 2b iii).

**Extended Data Fig. 1.**
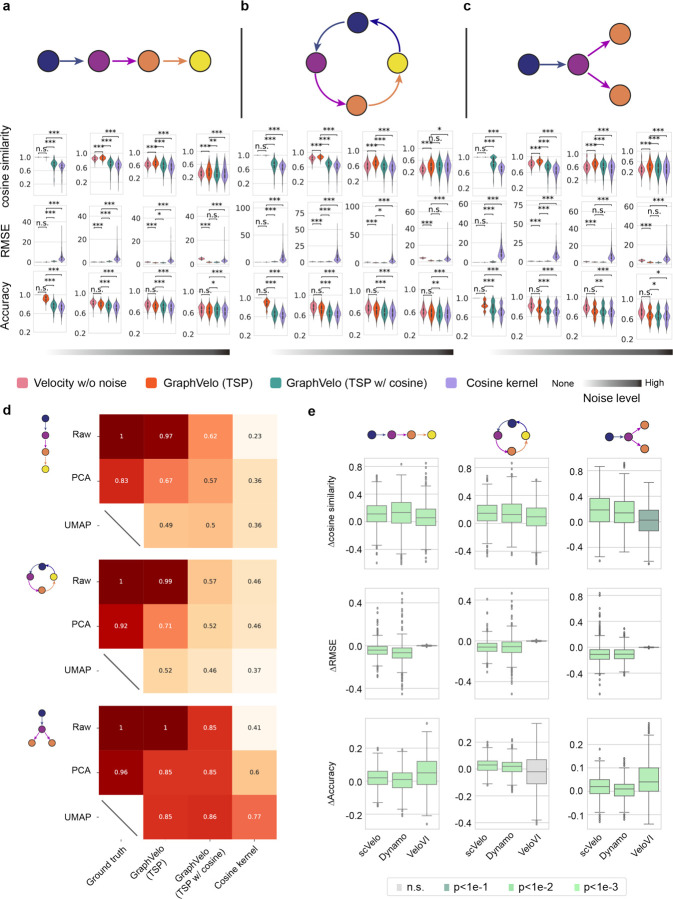
Refining RNA velocity from noisy simulation data or tradition splicing-based methods **(a-c)** Evaluation of on simulated scRNA-seq data under linear, cycling and bifurcating differentiation models with an increasing noise level in velocity vectors. **(d)** Heatmap of the correlation of cell speed calculated as the norm of velocity vector with respect to the full-dimensional RNA velocity and the norm of velocity vectors projected to PCA or UMAP space using GraphVelo, GraphVelo with cosine regularization, cosine kernel. **(e)** Boxplots of metric evaluations on GraphVelo correction to the original velocities estimated by scVelo, dynamo, and VeloVI. Box colors indicate the statistical test results for improvement relative to zero.

Next, we performed multifaceted evaluations on the ability of GraphVelo to robustly recover the transcriptional dynamics across a range of simulated datasets with different underlining phenotypic structures. We used dyngen^18^, a multi-modal scRNA-seq simulation engine, to generate gene-wise dynamics defined by gold-standard transcriptional regulatory networks (Methods). We generated simulated scRNA-seq data for networks with a variety of underlying linear, cyclic, and bifurcating topological structures, and recovered the vector field using GraphVelo-corrected velocity vectors (Fig. 2c–e). To comprehensively assess the outcome, we used three diverse metrics, Cosine similarity, root mean square error (RMSE), and accuracy to evaluate the correctness of velocity direction, magnitude, and sign, respectively, to benchmark the cosine kernel, TSP with (i.e., Eqn. 2 with b≠0) and without (Eqn. 2 with b=0) the cosine regularization term. By minimizing TSP loss, GraphVelo preserved both the direction and magnitude information (Fig. 2f–h). With an increase of noise level by adding Gaussian noise to the ground truth vectors, GraphVelo refined the distorted velocity and outperformed the cosine kernel projection consistently (Extended Data Fig. 1a–c). Next, we tested whether manifold constraints could preserve the speed of the cell progression across different representations. GraphVelo was able to scale velocity vectors between the original space and the PCA space, showing a high correlation with the ground truth, even as noise levels increased, whereas the cosine kernel failed (Extended Data Fig. 1d). The results on UMAP showed less agreement, which is not surprising. UMAP is a convenient representation for visualizing single cell data but not designed for representing quantitative cell state transition dynamics since UMAP is not a continuous transformation from the original gene space and cannot preserve local distances after projection.

To further explore whether GraphVelo could correct the RNA velocity estimated by the splicing kinetics, we took the velocity inferred using different packages (scVelo^2^, dynamo^7^ and VeloVI^5^) as input. The outcome of GraphVelo agreed significantly better with the ground truth compared to the raw input (Extended Data Fig. 1e), highlighting GraphVelo on improving the performance in both direction and magnitude of the velocity vectors significantly across all datasets.

### GraphVelo infers quantitative whole genome RNA velocity from a subset of genes with manifold-consistent RNA turnover kinetics

**Extended Data Fig. 2.**
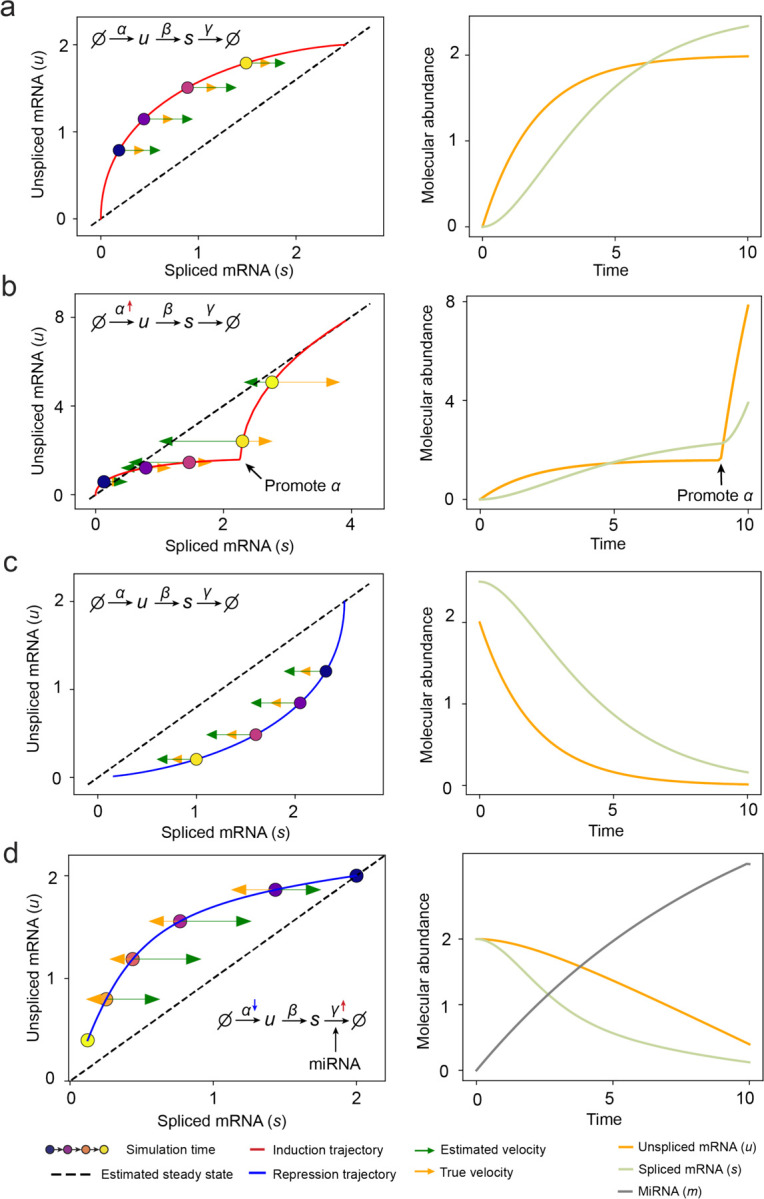
RNA velocity inference on simulation results of splicing kinetics with constant or changed rates. **(a)** Standard splicing kinetics along an induction trajectory in phase portrait and the reactions define how abundance levels of molecules change along simulation time. Right panel shows corresponding trajectories over time (same below). **(b)** Transcription burst along an induction trajectory in phase portrait. The transcription rate constant 𝜶 was promoted at specific time point. **(c)** Standard splicing kinetics along a repression trajectory. **(d)** Rapid time-varying degradation kinetics along repression trajectory. External signal promotes synthesis of microRNA, which enhances degradation of target mRNA resulting in a microRNA - dependent varying degradation rate “constant” γ, and inhibits the target gene via a decreasing α.

**Figure 3.**
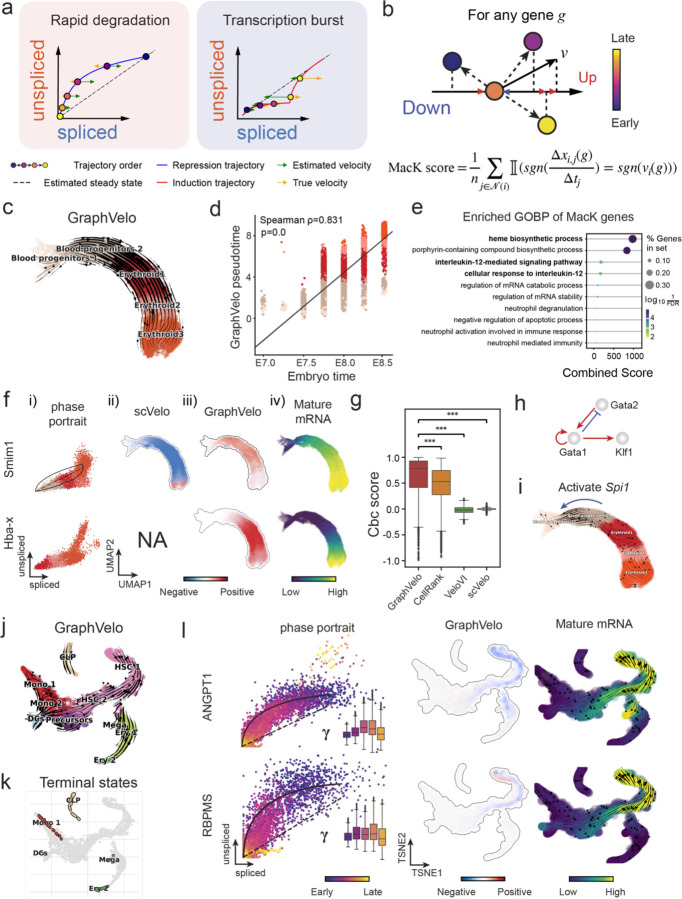
Delineating transcriptome-wise progression with manifold-consistent kinetic genes using GraphVelo **(a)** Schematitc of transcritional events mislead RNA velocity estimation in the phase portrait by standard approaches. **(b)** Schematic of manifold-consistent score calculation for robustly estimated velocity genes. **(c)** The projected velocity field from GraphVelo are consistent with the erythroid differentiation by using all highly variable genes. **(d)** The correlation between GraphVelo pseudotime and embryo time for erythroid lineage cells. Spearman correlation coefficients and *P* values are shown. **(e)** GO enrichment analyses of top ranked MacK genes. **(f)** Scatter plots of: i) phase portrait, ii) velocities estimated by scVelo, iii) refined velocities by GraphVelo, and iv) mature mRNA expression of transcription burst genes (*Smim1*, *Hba-x*). Cells were colored by cell type, corresponding velocity, and mature mRNA abundance, respectively, and visualized on the phase portrait and UMAP representation. **(g)** Cross boundary correctness calculated by the velocity input from GraphVelo, CellRank pseudotime kernel, VeloVI and scVelo, respectively. **(h)** Gene regulatory cascade unraveled by GraphVelo-based vector field analyses that drives cell lineage commitment. **(i)** Role of *Gata1* inhibitor TF *Spi1* in gastrulation erythroid maturation investigated through in silico perturbation analyses on GraphVelo-based vector field. **(j)** Velocities derived from GraphVelo for the branching lineage in the hematopoiesis development and projected onto a pre-defined TSNE embedding. Directions of the projected cell velocities on TSNE are in agreement with the reported differentiation directions. **(k)** Terminal states identified by CellRank based on Markov chain formulation. **(l)** Phase portrait, velocity estimated by scVelo, refined velocity by GraphVelo, and gene expression of mature mRNA of identified rapid degradation genes (*NPR3, ANGPT1*). The cells were colored by the dynamo vector field-based pseudotime in the phase portrait. The box plots showed cell-specific 𝜸 for cells divided into bins according to pseudotime ordering in the phase protrait.

Most RNA velocity methods are based on biophysical models of mRNA turnover dynamics with specific assumptions that may break down in certain cases^11^. For example, the splicing-based RNA velocity may have an erroneous sign for processes under active regulation on mRNA degradation or promotors switching between states with different transcription efficiency (Fig.3a, Extended Data Fig. 2). GraphVelo first uses the RNA velocities inferred from any method as input, then uses the velocities of high-confident genes to infer velocities of other genes. One can use several existing approaches on evaluating the confidence scores of inferred RNA velocity values of genes^7^. Alternatively, we identified a subset of Manifold-consistent Kinetics (MacK) Genes based on their agreement with additional prior knowledge or additional information acquired from other methods such as lineage tracing (Fig.3b),

We first applied GraphVelo to a mouse erythroid maturation dataset^19^. This study provided a transcriptional landscape of the erythroid lineage with well-documented differentiation trajectory during mouse gastrulation. Previous analyses have shown that the dataset contains genes with multiple rate kinetics, leading to erroneous prediction of the cell state transition direction^19,20^. We selected the top 200 out of 450 velocity genes as MacK genes, representing those with robustly estimated velocities (Methods). The projected vector field in UMAP showed consistency with prior knowledge in developmental biology (Fig.3c). We then used the corrected RNA velocities for Dynamo velocity field analyses. The vector field-based pseudotime accurately predicted the lineage with scRNA-seq data of temporal mouse embryos (Fig.3d).

**Extended Data Fig. 3.**
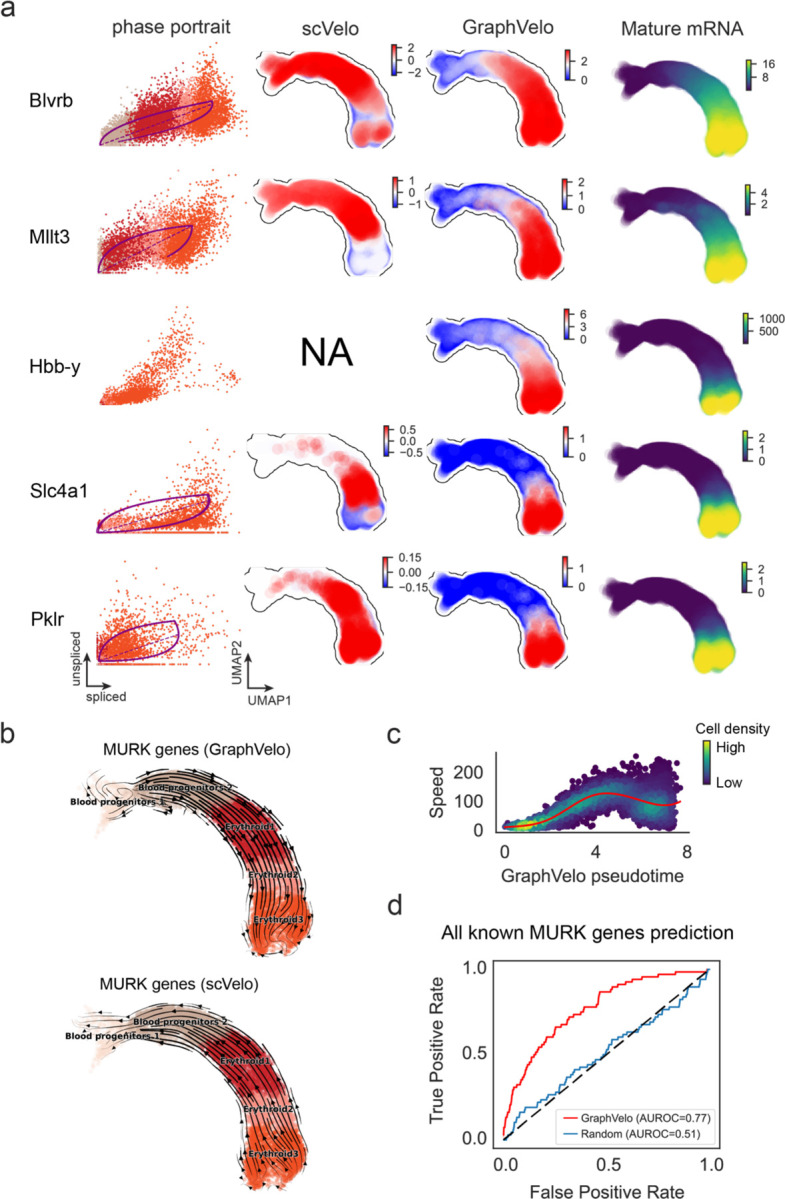
GraphVelo extrapolation of RNA velocities of an extended list of gene set in mouse erythroid maturation dataset. **(a)** Phase portrait, velocity estimated by scVelo, refined velocity by GraphVelo, and gene expression of mature mRNA of a selected set of genes. Cells were colored by cell type, corresponding velocity, and mature mRNA abundance, respectively, and visualized on the phase portrait and UMAP, respectively. **(b)** Velocities of MURK genes derived from GraphVelo and scVelo for gastrulation erythroid maturation cells projected to a predefined UMAP representation. Note that RNA velocity estimated by scVelo was in a reverse flow, possibly influenced by transcription burst events. **(c)** Cell speed distribution along the vector field pseudotime axis. Cells were colored by local density and red line indicates the fitted curve. **(d)** Receiver operating curve analyses of ranked acceleration genes predictions when using all known MURK genes as the gold standard in contrast to a random predictor. AUC, area under curve.

Previous studies identified multiple rate kinetics (MURK) genes showing transcription bursts in the middle of erythroid differentiation^20^. For example, *Smim1* and *Hba-x* are two MURK genes showing complex patterns of phase portrait (Fig. 3f i). Consequently, the RNA velocity of *Simi1* inferred with scVelo was negative along the most part of the developmental axis (Fig. 3f ii), contradicting the trend of increasing *Simi1* mRNA levels (Fig. 3f iv). For *Hba-x*, scVelo even failed to infer its RNA velocity. On the other hand, GraphVelo inferred velocities and predicted correct kinetic patterns of these genes (Fig. 3f iii). Similar performances have been observed in other MURK genes (Extended Data Fig. 3a). To examine the overall prediction of cell state transitions from transcription burst genes, we projected the MURK genes velocity inferred from GraphVelo and scVelo to the predefined UMAP. The velocities from GraphVelo but not scVelo correctly captured the directional flow of differentiation using only MURK genes (Extended Data Fig. 3b).

Next, we examined the inferred RNA velocities of an entire set of 2,000 highly variable genes. For each gene we calculated the cross-boundary correctness (CBC) score, which quantifies how likely, following its current velocity, a cell can develop to a target cell state^21^. GraphVelo outperformed several existing tools that only used hundreds of ‘well-estimated’ kinetics genes (Fig. 3g). Furthermore, we estimated the speed of cell state transition using the norm of velocity vector in high-dimensional space and identified the transcriptional surge stage (Extended Data Fig. 3c). We hypothesized that the MURK genes, which exhibited a sudden increase in transcription rate during this stage, were responsible for the sharp acceleration in cell state transition speed. Using dynamo, we estimated the acceleration derived from the GraphVelo vector field and found that the acceleration value, as the derivative of the velocity vector, demonstrated potential as a predictor for transcription burst genes (Extended Data Fig. 3d).

With the velocity estimation extended to the whole gene space, we were able to perform comprehensive mechanistic analyses on the entire genome spectrum. First, we calculated the MacK score for each gene using the corrected RNA velocities. We hypothesized that a gene with a higher MacK score indicated a better agreement between its RNA velocity vector and the developmental axis, suggesting that the gene as a potential lineage-driver gene. We ranked genes based on their scores and performed GO biological process enrichment analyses for the top genes. Indeed, the enriched processes were associated with erythropoiesis, including the heme biosynthetic process and interleukin-12-mediated signaling pathway^22^ (Fig. 3e).

**Extended Data Fig. 4.**
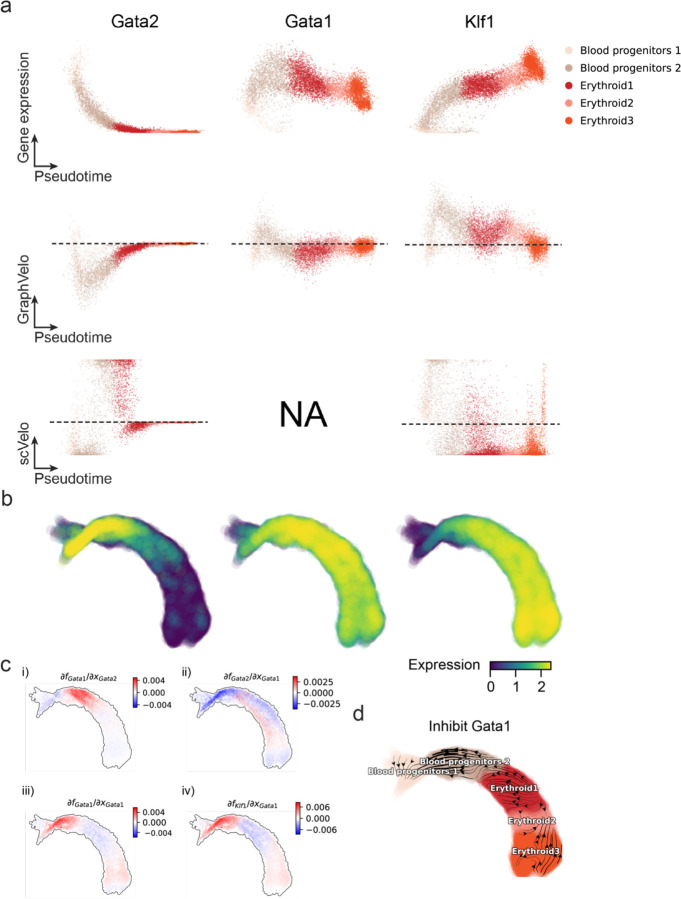
Regulatory cascades of driver TFs in mouse erythroid data. **(a)** Expression and corresponding velocities of *Gata2*, *Gata1*, *Klf1* along the vector field-based pseudotime in mouse erythroid data. **(b)** Gene expression of *Gata2*, *Gata1*, *Klf1* on the UMAP space. **(c)** Molecular mechanisms of driver TFs underlying erythroid lineage commitment based on Jacobian analyses. (i) *Gata2* activates *Gata1*. (ii) Repression of *Gata2* by *Gata1*. (iii) Self-activation of *Gata1*. (iv) *Gata1* activates *Klf1*. **(d)** In silico perturbation analyses on GraphVelo-based vector field to examine the role of *Gata1* in gastrulation erythroid maturation.

Next, we applied dynamo to perform differential geometry analyses of the vector field and mechanistically dissected the activation cascade of erythroid marker gene *Klf1* (Extended Data Fig. 4a&b). Jacobian analyses based on GraphVelo vector field revealed sequential activation of driver transcription factors (TFs) *Gata2*, *Gata1*, and *Klf1* during erythroid lineage differentiation, with *Gata1* subsequently repressing the expression of *Gata2* (Fig. 3h, Extended Data Fig. 4c)^7^. To further demonstrate the crucial role of transcriptional factor *Gata1* during erythropoiesis, we performed in silico genetic perturbation across all cells. Results showed that both inhibiting *Gata1* and upregulating the *Gata1* repressor *Spi1* lead to a reversal of normal developmental flow (Fig. 3i, Extended Data Fig. 4d). The above analyses collectively suggest that activation of *Gata1* in the blood progenitors biased its differentiation to erythropoiesis, agreeing with experimental reports^20^.

**Extended Data Fig. 5.**
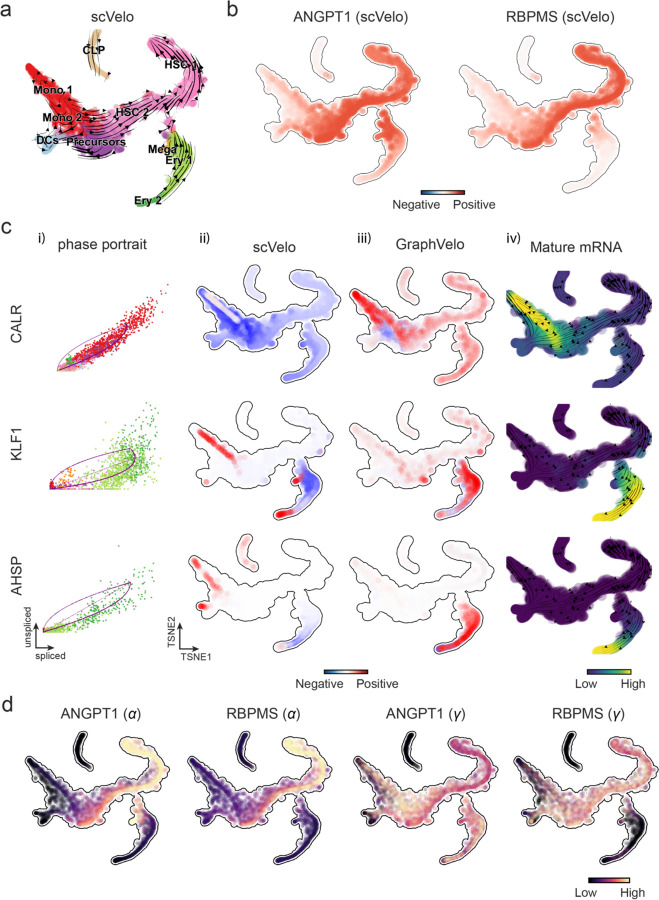
RNA velocity estimated by scVelo and refined by GraphVelo from a hematopoiesis dataset. **(a)** Velocities derived from scVelo in the hematopoiesis development and projected to a pre-defined TSNE embedding. **(b)** TSNE visualization of the RNA velocity estimated by scVelo of *ANGPT1* and *RBPMS* genes. **(c)** Scatter plots of: i) phase portrait, ii) velocities estimated by scVelo, iii) refined velocities by GraphVelo, and iv) mature mRNA expression of transcription burst genes (e.g. *CALR*, *KLF1* and *AHSP*). Cells were colored by cell type, corresponding velocity, and mature mRNA abundance, respectively, and visualized on the phase portrait and TSNE, respectively. **(d)** Cell-specific transcription rate constant 𝜶 and degradation rate constant 𝜸 of rapid degradation genes *ANGPT1* and *RBPMS* visualized on the TSNE space, which is consistent with simulation result in Extended Data Fig. 2d.

To further evaluate GraphVelo, we tested the method on another dataset of human bone marrow development^23^. This developmental process has complex progressions from hematopoietic stem cells (HSCs) to three distinct branches: erythroid, monocyte, and common lymphoid progenitor (CLP). Again, we used the top 100 out of 454 velocity genes as MacK genes to predict the velocities of 2,000 highly variable genes. The GraphVelo velocity field accurately recovered the fate of cells on the sophisticated transcriptional landscape in contrast to scVelo(Fig. 3j and k, Extended Data Fig. 5a). By combining the likelihood estimated by scVelo with the MacK score, we identified rapid degradation and transcription burst genes whose dynamics deviated from the RNA velocity assumptions (Extended Data Fig. 5b&c). *ANGPT1* and *RBPMS* are two examples which were overall highly expressed in the progenitors and decreased quickly along the trajectories (Fig. 3l), reminiscent of what is shown in Figure 3a. These genes misled RNA velocity inference with scVelo assuming a constant degradation rate constant. GraphVelo revealed the degradation wave along the differentiation path with a cell context-specific transcription rate $\alpha =u+\frac{du}{dt}$ and degradation constant $\gamma =\left(u-\frac{ds}{dt}\right)/s$ (Fig. 3l, Extended Data Fig. 5d), consistent with simulation result and reports on regulation of *ANGPT1* mRNA by microRNAs such as *miRNA-153-3p*^24^ (Extended Data Fig. 2d).

### GraphVelo reconstructs host–pathogen transcriptome dynamics from infection trajectory

**Figure 4.**
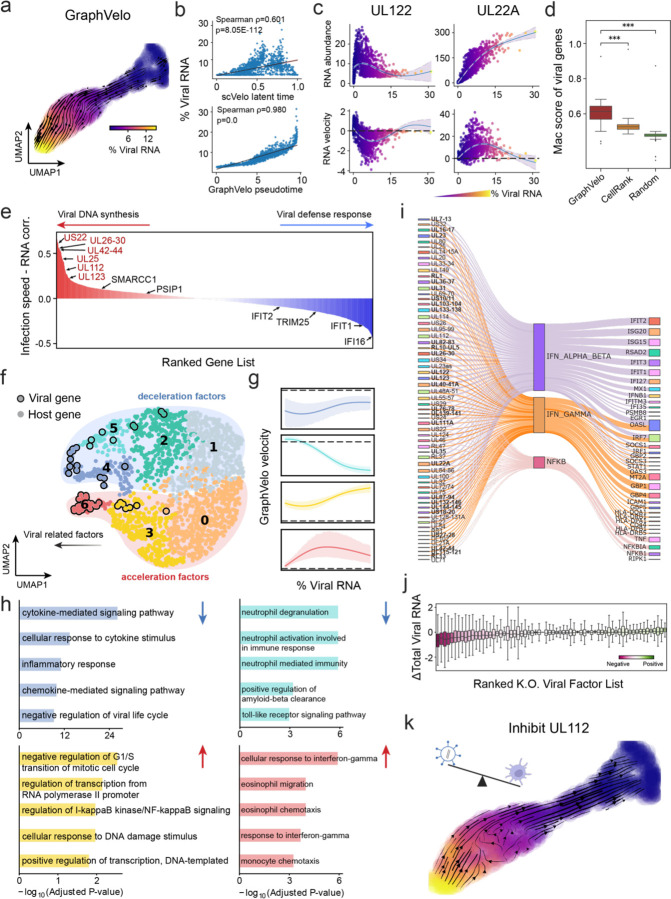
Using GraphVelo velocities to infer host-virus infection trajectory and identify host-pathogen interactions **(a)** GraphVelo velocity field colored by the percentage of viral RNA within a single cell. **(b)** Correlation between viral RNA percentage and pseudotime inferred by scVelo or GraphVelo. Spearman correlation coefficients and *P* values were shown. **(c)** Viral RNA velocities infered by GraphVelo along the viral RNA percentage axis. The black dot line highlights the zero velocity. **(d)** Boxplot summarizing the Mac scores of all viral genes calculated by GraphVelo, CellRank pseudotime kernel and random predictor. **(e)** Correlation between viral infection speed and RNA abundance. Genes were ranked by Spearman correlatioin coefficients. Host and viral genes that contribute to viral DNA synthesis were marked in the left side and those contribute to viral defense response were marked in the right side. Viral genes were highlighted in red. **(f)** UMAP representation of host and viral genes with distances defined by their dynamic expression patterns along the viral RNA percentage axis. **(g)** Example dynamic expression patterns within specific clusters (Leiden4, 5, 3, 6 from top to bottom) along the viral RNA percentage axis. Zero velocity was highlighted by black dot line. **(h)** GO enrichment of each cluster in (g). **(i)** Top host genes inhibited by each viral factor based on dynamo Jacobian analyses. Host effectors were organized by their involved pathways. **(j)** Dynamo prediction of total viral RNA change in response to in silico viral factor knockout. Viral factors were ranked by the mean of total viral RNA changes. **(k)** Vector field change resultant from infinitesmal inhibition of *UL122* during the viral infection process.

Continuous battle between human immune surveillance and viral immune evasion takes place in the host cell system. Currently, scRNA-seq provides a massive and parallel way of assessing the outcome of both host and viral transcripts, unraveling the delicate inherent dynamic of a virus-host system^25,26^. While existing splicing-based methods can robustly estimate RNA velocity of host coding genes, it is infeasible to infer the viral dynamics in the transcriptomic level due to alternative splicing and the lack of intron in the viral genome. GraphVelo enables inference of the velocities of virus RNA abundance based on the kinetics of host transcripts velocities. We analyzed a human cytomegalovirus (HCMV) viral infection dataset to learn viral transcriptomic kinetics in monocyte-derived dendritic cells (moDCs)^27^. The result of GraphVelo unraveled how virus infection progressed along the transcriptional continuum (Fig. 4a). The velocity vectors pointed to directions consistent with an increasing trend of the percentage of viral RNAs in a single cell, which served as an inherent indicator of the infection time course^28^. Compared to that with the raw RNA velocities from scVelo, the vector field-based pseudotime calculated using GraphVelo-corrected RNA velocities showed higher consistency with the (pseudo)temporal progression of viral accumulation as reflected by viral RNA percentage (Fig. 4b).

**Extended Data Fig. 6.**
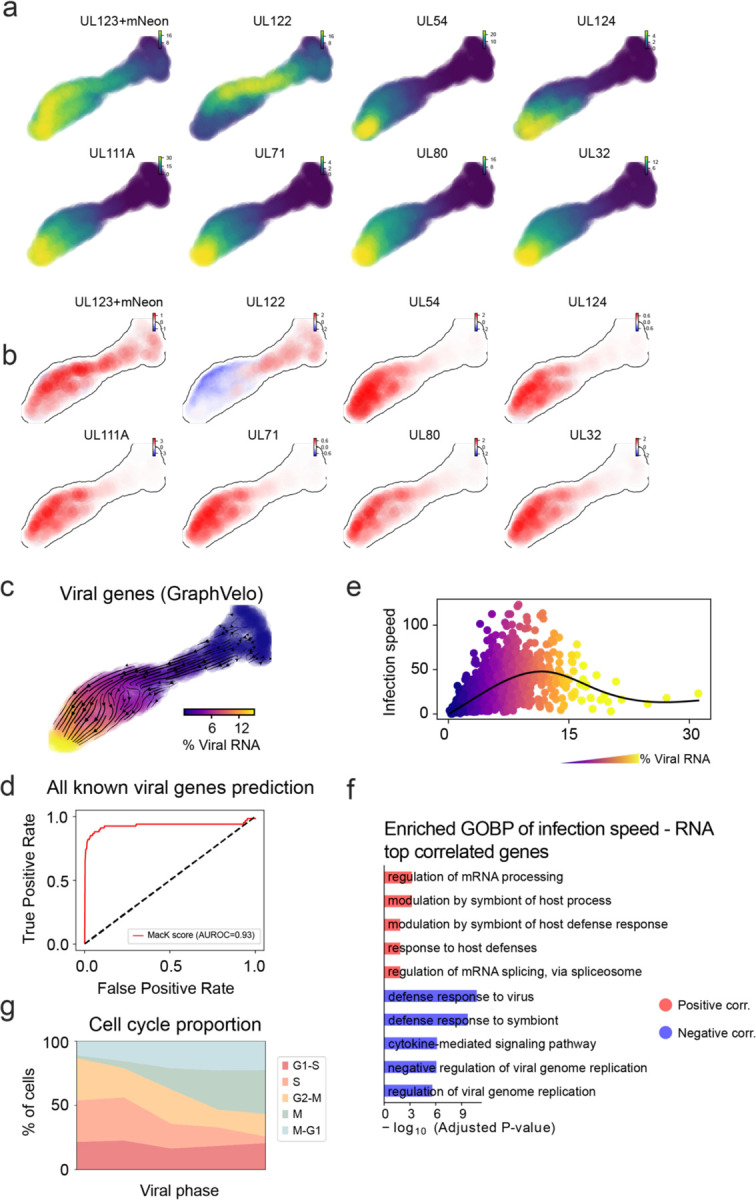
Dynamic patterns of gene trajectories during HCMV infection. **(a)** Gene expression of selected viral factors on the UMAP space. **(b)** Corresponding velocities inferred by GraphVelo for viral factors in (a). **(c)** Viral RNA velocities derived from GraphVelo for infected cells, projected to the UMAP representation only using viral genes. **(d)** Receiver operating curve analyses of MacK scores when using all detected viral genes as the gold standard. **(e)** Viral infection speed versus viral load. Cells were colored by the percentage of viral RNA. **(f)** GO enrichment analyses of top host genes correlated with infection speed. **(g)** Shifts in cell proportions across different cell cycle phases along the viral phase trajectory. Cells were divided into bins according to viral RNA percentage. The result indicates a decreasing S phase population corresponding to infection-driven transcriptional alterations of the cell cycle.

Examination of individual genes revealed that the RNA velocities consistently predicted the trend of the mRNA expression level change with increasing virus load (Fig. 4c, Extended Data Fig. 6a&b). Most virus genes started with a fast-increase phase, and the expressions of some genes (e.g. *UL22A*) gradually saturated at high virus load, together with the corresponding RNA velocities approaching zero. One exception is *UL122*, whose expression profile increased first then decreased to a steady state level lower than the peak value. This overshooting is characteristic of a negative feedback network structure^29^. Indeed a recent study reported that *UL122* negatively regulates its own promotor^30^. Furthermore, comparison of MacK scores across GraphVelo, the CellRank pseudotime kernel, and randomized prediction showed that GraphVelo viral RNA velocities aligned with the transcriptome gradient of viral load (Fig. 4d). The MacK score also served as a reliable predictor for dynamics-driving factors, specifically viral genes in this case (Extended Data Fig. 6d).

We quantified the velocity norm of all viral factors as infection speed, and observed that transcription of viral factors was significantly restricted initially, then gradually increased along the trajectory (Extended Data Fig. 6e). Interestingly, we found most genes positively correlated to the infection speed were related to viral DNA synthesis, while those negatively correlated to the infection speed were engaged in host viral defense response^31^(Fig. 4e, Extended Data Fig. 6f).

### GraphVelo identifies host genomic response modules and predicts host-virus gene interactions

With GraphVelo inferred RNA velocities, we probed the dynamics upon lytic infection and the complex interplay between host and viral functional genomes. By fitting the GraphVelo velocity trends along the viral load axis, we identified genes with similar kinetic patterns (Figure 4c). Using the smoothened velocity trends to calculate the distance as a measure, we clustered individual genes into seven major genomic modules and visualized them on the UMAP space (Fig. 4f). Not surprisingly, viral genes were concentrated in several enclosed regions, indicating that they formed distinct functional genomic modules during the lytic cycle^28^. The genes showed two major distinct dynamical features: acceleration and deceleration along the viral load axis (Fig. 4g).

To systematically investigate whether the dichotomy between host kinetics genes and viral genes share similar dynamics, we performed gene functional enrichment analyses of host genes residing close to the viral gene clusters. Genes located in the deceleration part were associated with repression of viral genome replication, including negative regulation of viral life cycle and known restriction factors in antiviral responses activated in DCs such as inducing cytokine and chemokine responses as well as interaction with neutrophil^32,33^. Similarly, the toll-like receptor signaling pathway, required for antiviral defense, was arrested^34^. Neutrophil related processes, which typically cooperate closely with DCs to modulate adaptive immune responses^35^, were suppressed. Therefore, the deceleration part showed how critical set of host factors were silenced by viral entry to achieve immune evasion. Meanwhile, the acceleration groups demonstrated how viruses hijacked the host cell endogenous cellular programs for virus replication. Notably, the pathways related to viral genome replication were triggered, promoting DNA replication and transcription, such as negative regulation of G1/S transition of mitotic cell cycle^36^, cellular response to DNA damage stimulus^37^ and regulation of transcription from RNA polymerase II promoter^38^. Cells showed a shift towards a transcriptional signature resembling the G1 phase (Extended Data Fig. 6g), agreeing with previous report on HCMV infection^39^. Along the infection process, antiviral interferon (IFN)-gamma response of moDC cells was first activated then suppressed. These results highlighted organized and antagonistic strategies adopted by both host cells and viruses during their tug-of-war on survival and proliferation.

To investigate the crosstalk between host and viral factors systematically in depth, we performed dynamo Jacobian analyses. We scanned the entire spectrum of viral genome and delineated how the HCMV factors silence IFN and NFκB signaling (Fig. 4i). A large proportion of the identified viral factors functioned in immune evasion against host cells, a finding supported by several recent studies^40–42^. In silico virus-directed knock out experiments revealed altered accumulation patterns of viral transcripts (Fig. 4j). Notably, inhibition of *UL112*, which ranked first in our analyses, led to a qualitatively distinct trajectory. These results highlight the multifunctional *UL112* locus in the viral genome as a potential target for antiviral intervention^28,43^. The analyses demonstrated potential usage of GraphVelo-inferred velocities on understanding the interactions between viral and host factors, as well as the effects of perturbations on infection, and for designing antiviral interventions^28^.

### GraphVelo permits multi-omics velocity inference and chromatin dynamics analyses

**Figure 5.**
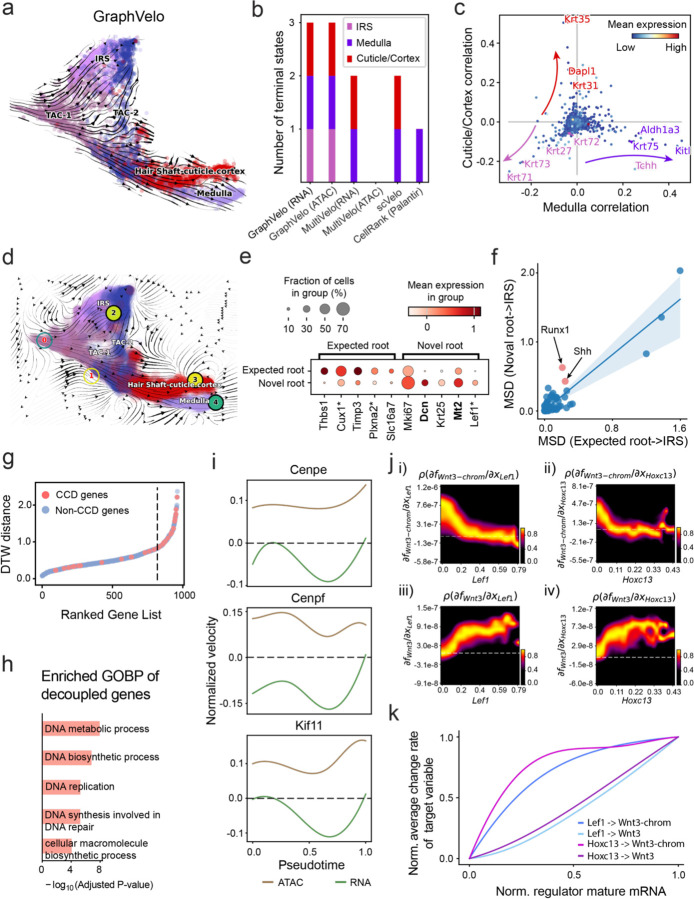
Inferring epigenome and transcriptome consistent dynamics in mouse hair follicle development using GraphVelo multiomic velocities. **(a)** GraphVelo velocity fields colored by cell macrostates. **(b)** Number of terminal states predicted by CellRank using velocities inferred with different methods. **(c)** Driver genes along multiple lineages identified through CellRank. **(d)** Topological analyses of GraphVelo vector field identified novel root cells and attractors residing in three terminal states. **(e)** Expression levels of marker genes in novel root cells and expected root cells. Markers identified by Ma et al.^44^ were highlighted with stars and newly identified markers were highlighted in bold. **(f)** Regression results of MSD values along the transition path from the expected root or novel root to IRS. Two genes *Runx1* and *Shh* genes with large MSD originating from the novel root were highlighted. **(g)** DTW distance between RNA velocity and chromatin velocity of individual genes. CCD genes were colored in red. The dotted line indicates the elbow point separating the decoupled genes from the rest. **(h)** GO enrichment of decoupled genes in (g). **(i)** Line plot of nomarlized RNA and chromatin velocity along pseudotime for genes predicted by GraphVelo to have notable decoupling patterns. Chromatin velocity trends were colored as brown and RNA velocity trends were colored as green. **(j)** Heatmaps of jacobian element distribution along the axis of regulator RNA abundance of four regulator effector circuits: i) Lef1 versus Wnt3 chromatin openess. ii) Hoxc13 versus Wnt3 chromatin openess. iii) Lef1 versus Wnt3 transcription. iv) Hoxc13 versus Wnt3 transcription. **(k)** Effective dose-response curves obtained from integrating the averaged Jacobian elements over the corresponding normalized regulator mature mRNA regulator level in (j).

The molecular anatomy during cell development is composed of multiple layers, and how different layers coordinate on regulating gene expression is a fundamental problem. For example, the anagen hair follicle features distinct lineages branching from a central population of progenitor cells. Ma et al^44^. used SHARE-seq to capture both transcriptome and epigenome information simultaneously for the lineage commitment process from transit-amplifying cells (TACs) to the inner root sheath (IRS), cuticle layer, and medulla. Taking robustly estimated genes selected following dynamo criteria (Methods), we further refined the RNA velocities of these genes through tangent space projection and obtained the chromatin open/close dynamics from the corresponding scATAC data with GraphVelo. The resultant vector field in the combined transcriptome-epigenome space reconstructed the correct multilineages differentiation paths during the anagen phase (Fig. 5a).

**Extended Data Fig. 7.**
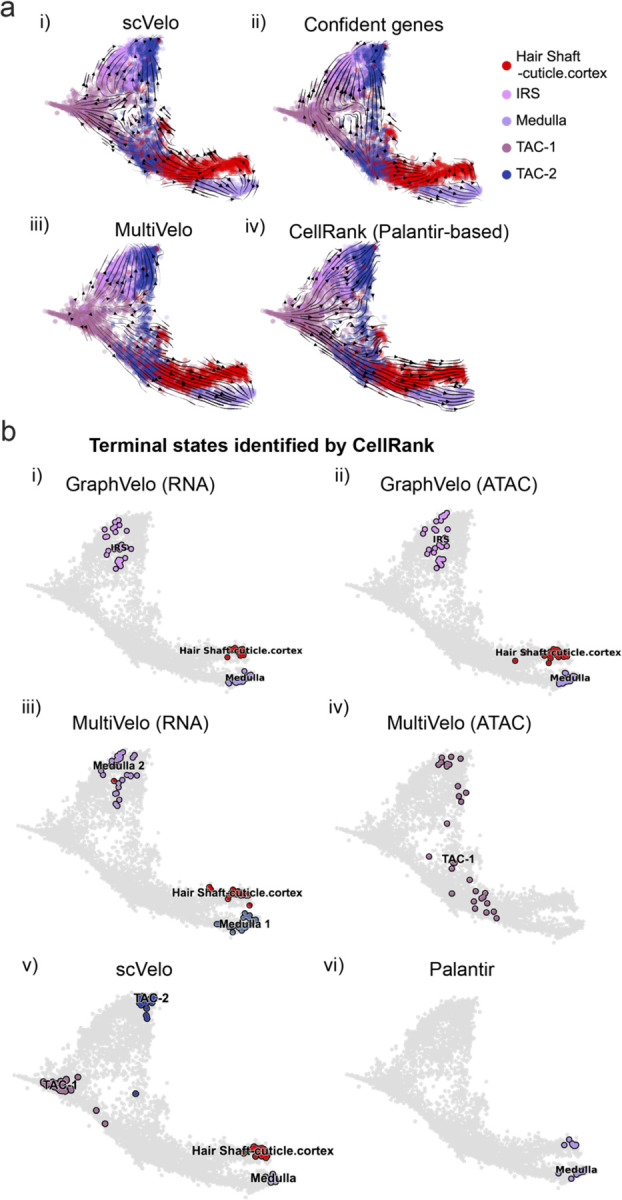
GraphVelo estimation of modality-consistent vector field based on multi-omics velocities. **(a)** Velocity field projected to the pre-defined UMAP representation with different methods or gene sets: i) scVelo RNA velocity based on all velocity genes; ii) scVelo RNA velocity based on confident velocity genes filterred by dynamo criteria; iii) MultiVelo RNA velocity based on all velocity genes; iv) CellRank pseudotime kernel based on Palantir pseudotime. **(b)** Terminal states identified by CellRank using different representation and corresponding velocity: i) GraphVelo RNA velocity; ii) GraphVelo chromatin velocity; iii) MultiVelo RNA velocity; iv) MultiVelo chromatin velocity; v) scVelo RNA velocity; vi) Palantir-based pseudotime kernel.

**Extended Data Fig. 8.**
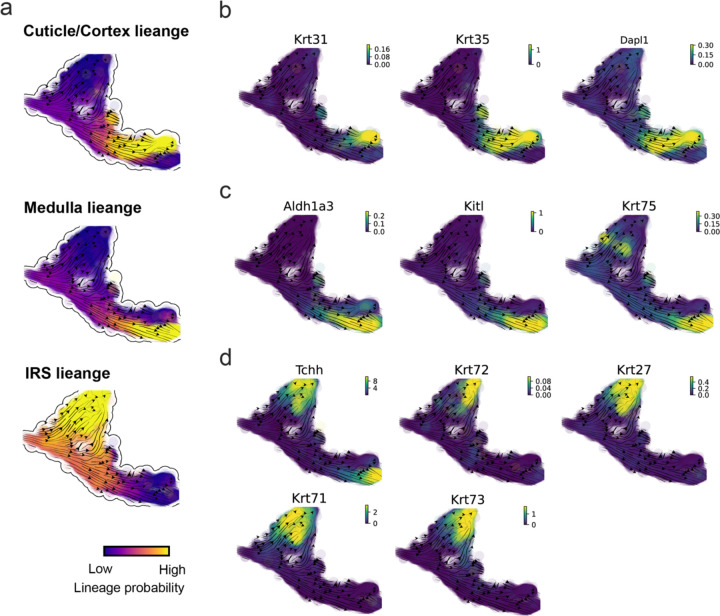
Reconstructed lineage commitment during mouse hair follicle differentiation using GraphVelo velocities. **(a)** Lineage commitment probablity of each terminal cell type on UMAP vector field. **(b-d)** Gene expression distribution of markers for cuticle/cortex, medulla and IRS lineages on the UMAP vector field.

To test the consistency of dynamics across different modalities, we performed CellRank terminate stage analyses^45^ from the refined velocity vectors. Using GraphVelo velocities of either the RNA modality or the ATAC modality, we accurately estimated three diverse terminal stages (Fig. 5b). For comparison, we also performed similar analyses using MultiVelo, scVelo with all velocity genes or robustly estimated genes in above GraphVelo studies and pseudotime-based vector field inferred by CellRank. The 2-D projection of these vector field functions also exhibited seemingly correct velocity flow direction (Extended Data Fig. 7a). However, none of them captured the cell fate commitment based on coarse-grained transition matrix (Fig. 5b, Extended Data Fig. 7b). Notably, the results from the RNA modality and the ATAC modality of MultiVelo gave inconsistent results. Based on the GraphVelo-corrected velocities, we identified the top-correlating genes towards individual terminal populations which showed agreement with previous study^46^ (Fig. 5c, Extended Data Fig. 8).

**Extended Data Fig. 9.**
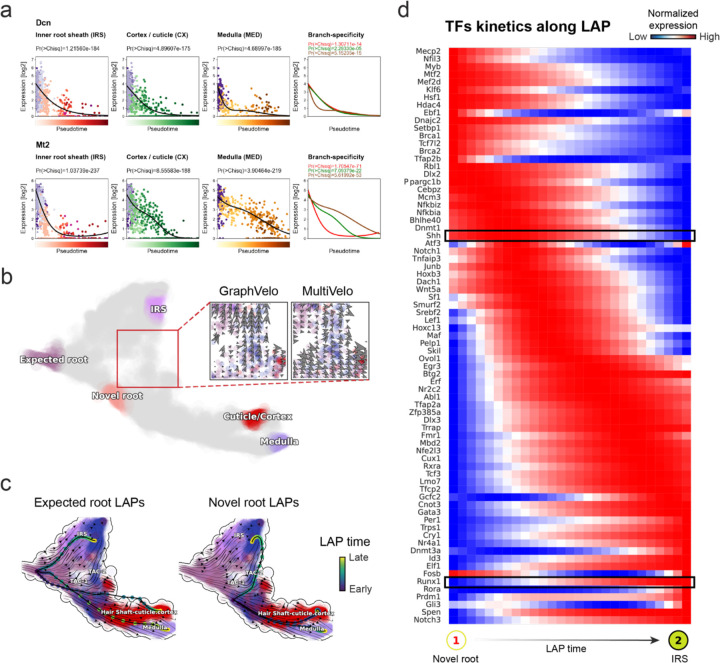
Novel root identified with multi-omic vector field from GraphVelo velocities. **(a)** *Dcn*, *Mt2* expression dynamics during anagen hair follicle keratinocytes^46^. **(b)** Regions identified by topological analysis. Insert are ummarized cell-state transition vectors calculated by GraphVelo and MultiVelo along the path from the novel root to IRS and projected onto the UMAP representation. **(c)** Predicted developmental LAPs from expected root or novel root to to each of the terminal cell types in the UMAP embedding. Color of the node along the paths indicates the LAP transition time. **(d)** TF expression profiles along the LAP from novel root to IRS. *Shh* decays, alongside the induction of the *Runx1* gene.

Next, we conducted differential geometry analyses based on the composite GraphVelo vector field. We identified novel root cells, which were also characterized by chromatin potential (Fig. 5d)^44^. These novel root cells expressed distinct marker genes compared to the expected root cells using the Wilcoxon test (Fig. 5e). Moreover, we unraveled differentially expressed markers identified by the original study^44^, as well as new differentiation-potent genes and validated their initiation properties in another transcriptome dataset (Extended Data Fig. 9a)^46^. To further investigate how these two distinct groups of root cells convert to other cell types, we performed the least action path (LAP) analyses between different cell phenotypes. The expected and novel root cells converted to the IRS terminal state following two distinct LAP paths in the vector field (Extended Data Fig. 9b&c). The two paths revealed different temporal change patterns of transcription factor expression profiles (Extended Data Fig. 9d). We calculated the mean squared displacement (MSD) for every transcription factor to explore the dynamics of TFs along the path from novel root to IRS. The result demonstrated that the fate conversion by novel root was mediated by the Shh-Runx1 signaling axis (Fig. 5f, Extended Data Fig. 9d), which has been demonstrated in human embryonic stem cells^47^ and is crucial for hair development^48^. In summary, GraphVelo unraveled that multiple molecular mechanisms orchestrated hair follicle morphogenesis.

**Extended Data Fig. 10.**
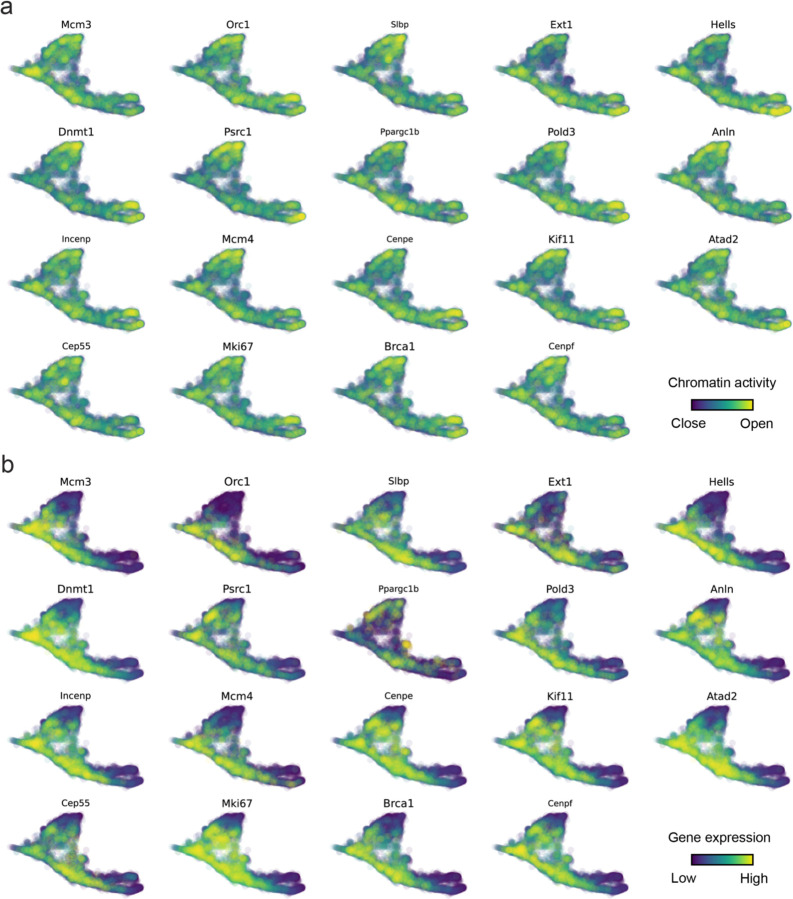
Genomic patterns of decoupled CCD genes. **(a)** Chromatin activity of decoupled CCD genes. **(b)** Gene expression of decoupled CCD genes.

**Extended Data Fig. 11.**
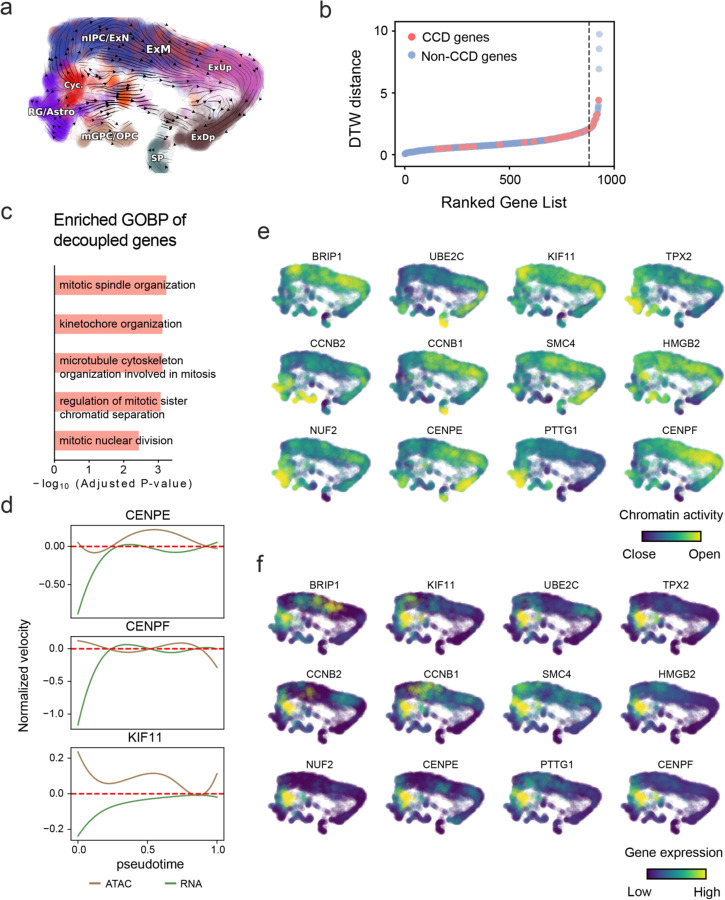
GraphVelo inferrence on epigenome and transcriptome decoupling dynamics in fetal human brain. **(a)** GraphVelo velocity field colored by cell macrostates. **(b)** DTW distance between RNA velocity and chromatin velocity of individual genes as a measure of the coupling/decoupling status. CCD genes were colored in red. The dotted line indicates the elbow point, with the decoupled genes on its right. **(c)** GO enrichment of decoupled genes in (c). **(d)** Line plot of nomarlized RNA and chromatin velocity along dynamo vector field pseudotime for genes predicted by GraphVelo to have notable decoupling patterns. Chromatin velocity trends were colored in brown and RNA velocity trends were colored in green. **(e)** Chromatin activity of decoupled CCD genes. **(f)** Gene expression of decoupled CCD genes.

With available chromatin velocity and RNA velocity, we set to quantify the coupling/decoupling relationships between chromatin structure and gene expression for each gene (see Methods). We used DTW distances between velocities from different omics layers to quantify the similarity between temporal patterns of these two modalities for each gene, and a higher value indicates higher similarity. Using the elbow of the ranked distance curve as a cutoff we identified genes that showed decoupled transcription and chromatin structure dynamics. These decoupled genes had an accumulation of cell cycle-dependent (CCD) genes found in previous study^49^ (Fig. 5g). This group of genes showed strong involvement in cell cycle-related processes, as indicated by GO enrichment analyses (Fig. 5h). Close examinations indicated that the transcription of cell cycle related genes decreased along the differentiation path, while the chromatin structure at the corresponding loci remained open (Fig. 5i, Extended Data Fig. 10). To validate this hypothesis, we further applied GraphVelo to a recently published 10x Multiome dataset from developing human cortex^50^ (Extended Data Fig. 11a). Following the same analyses, we identified decoupled genes and found out that most of these genes were related to cell cycle (Extended Data Fig. 11b–f). which has also been reported in a previous MultiVelo study^13^.

**Extended Data Fig. 12.**
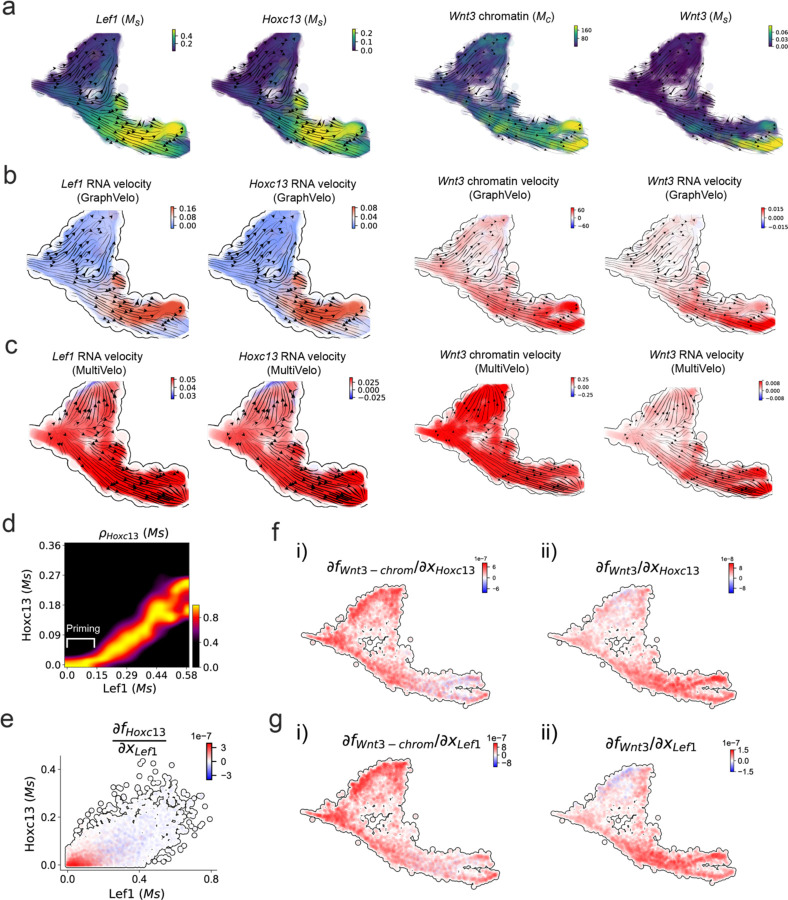
GraphVelo prediction on the regulatory mechanisms of Lef1-Hoxc13-Wnt3 circuit during mouse skin development. **(a)** Distributions of Lef1 expression, Hoxc13 expression, Wnt3 chromatin openess and Wnt3 expression, respectively, on the projected UMAP vector field. **(b)** Velocities of Lef1 RNA, Hoxc13 RNA, Wnt3 chromatin and Wnt3 RNA inferred by GraphVelo, visualized on the projected UMAP vector field. **(c)** Velocities of Lef1 RNA, Hoxc13 RNA, Wnt3 chromatin and Wnt3 RNA inferred by MultiVelo, visualized on the projected UMAP vector field. **(d)** Expression of Hoxc13 gene versus Lef1 expression. Lef1 expression primes the activation of Hoxc13. **(e)** Cell-wise Jacobian analyses of Lef1-Hoxc13 activation cascade. **(f)** Jacobian analyses of regulatory interactions between potential PTF Hoxc13 and i) Wnt3 chromatin openess or ii) transcription.. **(g)** Jacobian analyses of regulatory interactions between PTF Lef1 and i) Wnt3 chromatin openess or ii) transcription.

We further performed dynamo differential geometry analyses on the composite transcriptome-chromatin vector field. One intriguing phenomenon observed in lineage dynamics is that Lef1 and Hoxc13 are the driver TFs correlated with domains of regulatory chromatin (DORCs) of Wnt3^44^. Differential geometry analyses on the composite vector field can go beyond correlation analyses and provide an underlying casual mechanism. As a prerequisite for such analyses, GraphVelo inferred RNA and chromatin velocities of the three genes correctly predicted the trend of change of mRNA and ATAC-seq counts (Extended Data Fig. 12a&b), in contrast to the performance of MultiVelo (Extended Data Fig. 12c). Then, Jacobian analyses on the GraphVelo vector field confirmed that priming activation of Lef1 subsequently activated the Hoxc13 TF^51^ (Extended Data Fig. 12d&e). Both Lef1 and Hoxc13 were found to activate the Wnt3 target gene, initiating lineage commitment (Extended Data Fig. 12f &g). To quantitatively understand how the two TFs affect Wnt3 chromatin structure and transcription, we plotted the response heatmap to reflect the distributions of Jacobian elements versus the abundance of mature mRNA for each TF (Fig. 5j). The two terms ∂fWnt3−chrom∂xLef1 and ∂fWnt3−chrom∂xHoxc13 started with positive values at low concentrations of TF mRNA copy numbers then decreased to zero, indicating that increasing the level of either TF lead to further opening of the Wnt3 chromatin region, and the effect saturated at high TF expression. The other two term and ∂fwnt3∂xLef1 and ∂fwnt3∂xHoxc13 increased with the TF levels, indicating that these two TFs also activated Wnt3 transcription. Through integrating the Jacobian elements over regulator expression changes, we obtained the effective dose-response curves obtained (see Methods), which revealed more transparently the TF dose lag between opening the target chromatin region and initializing transcription (Fig. 5k). Our results illustrated the sequential events that these two driver TFs acted as pioneer transcription factors (PTFs) to initiate local chromatin opening and then activated the transcription of Wnt3. Interestingly, computational methods and experimental research confirmed that Lef1 was identified as a nucleosome binder and exhibited diverse binding patterns across various cell lines^52^. The Hox family of TFs has also been shown to have the capacity to bind their targets in an inaccessible chromatin context and trigger the switch to an accessible^53^, consistent with our analyses that Hoxc13 shared a similar regulation mechanism with known PTF Lef1.

## Discussion

In this work, we provided a general framework that extends the RNA velocity and related approaches into various data modalities such as proteomics, spatial genomics, 3d genome organization, and imaging data that these approaches originally do not apply. We validated GraphVelo using various in vivo cellular kinetics models, confirming its efficacy and robustness in handling complex and noisy multi-modal data. GraphVelo can be seamlessly integrated with broad downstream analyses, such as Dynamo continuous vector field analyses, Markovian analyses using Graph Dynamo or CellRank.

## Extended Data

## Data Availability

Python package GraphVelo can be accessed from https://github.com/xing-lab-pitt/GraphVelo.
